# Remarks on Mr. Crowfoot's and Dr. Langslow's Statements of a Case of Apoplexy, with Incidental Observations on That Disease
†Vide Medical and Physical Journal, No. XXXIV.


**Published:** 1802-02

**Authors:** G. Wilkinson

**Affiliations:** Corresponding Member of the Medical Society of London, Member of the Royal College of Surgeons, and Honorary Member of the Chirurgo Physical Society of Edinburgh


### *Remarks on* Mr. Crowfoot's *and* Dr. Langslow's *Statements of a Case of Apoplexy*, † *with incidental Observations on that Disease.*

*By* G. Wilkinson, *Corresponding Member of the Medical Society of London, Member of the Royal College of Surgeons, and Honorary Member of the Chirurgo Physical Society of Edinburgh.*

---

### To Dr. BRADLEY.

Sir,

THE Statements of Mr. Crowfoot and Dr. Langſlow of a caſe, or ſuppoſed caſe, of Apoplexy, now before the public, appears, in my opinion, to favour more of perſonal animoſity than that of a candid and liberal diſquiſition into the nature of the complaint. Both parties are equally tenacious of their opinions, and theſe ſeem as diametrically oppoſite. But, as the Editors of the Medical Journal juſtly obſerve, that many of their readers may be divided in their opinions on this important diſeaſe, and that a knowledge of its true cauſes, and the moſt rational mode of treatment, cannot be too ſoon, or too widely diffuſed, and that they have ſubmitted it to their Readers, with a view of collecting their ſentiments—I beg leave, with all due deference and reſpect to the generality of profeſſional readers, and to Mr. C. and Dr. L. (to neither of whom I am perſonally known) to offer ſome few Remarks on the Caſe, as related by both of them; and as opinions ever will vary in diſeaſes not perfectly aſcertained, or clearly underſtood, ſubmit what are my general ſentiments on the nature and treatment of this important and often fatal diſeaſe, to the animadverſions and obſervations of better judges than myſelf; being fully perſuaded, that whatever new light may be thrown on a ſubject that ſtill appears involved in doubt and obſcurity, will be thankfully received.

Different as the Statements of this Caſe may appear, it ſeems however agreed, that it was an Apoplexy, although neither of the gentlemen ſaw the patient in the fit. When I ſay this, I do not mean to diſpute its reality, nor diſtruſt the profeſſional abilities of Mr. Primroſe, the gentleman firſt called in, who conſidered it as ſuch. The term Apoplexy was applied to it, and of courſe a difference of opinion not only took place with reſpect

---

† Vide Medical and Phyſical Journal, No. XXXIV.

refpect to the nature of this difeafe, but likewife the mode of treatment neceffary to be adopted. This, as far as it can be collected from both Statements, appears to be the matter in difpute.

In Mr. C's account, it appears that this lady, 59 years of age, was feized immediately after dinner; but neither Mr. C. nor Dr. L. have given us any intimation of the patient's ftate of health prior to this fudden attack.

Dr. L. however, by way of exultation, has thrown fome light on this affair in his anfwer to Mr. C. at the time of writing it,* by faying that his patient, at that period, was "in more perfect health than fhe had been for feveral weeks *previous* to her attack." From hence it muft be inferred, that fhe was then in an *imperfect* ftate of health before the fit took place. If the digeftive organs were in fault, which there is fome reafon to fuppofe might be the cafe; or the food which fhe had then eaten was improper; or, in other words, fhe had indulged her appetite too freely in any particular fort of diet, we fhall be enabled to develope, in fome meafure at leaft, one of the obvious caufes of this attack.

In the majority of cafes of this fort that ufually occur, it will be found, as Doctor Heberden juftly obferves,† that they chiefly "attack perfons who are paft the meridian of life, and frequently fuch as are at leaft upon the verge of old age. They are often the diftempers of perfons worn out with cares, and difeafes, and time; and feldom of the young, vigorous, and of the fubjects of inflammatory diforders."

It will alfo be found, that they often attack perfons after a full meal, and ufually thofe who are accuftomed to indulge in free living. It was in cafes of this fort, if I underftand rightly, that the late worthy Dr. Fothergill (whofe mode of treatment Dr. L. has fo arrogantly criticifed‡) trufted to evacuations by the ftomach and inteftines. How far he might be juftified in reftricting the ufe of the lancet, may not be eafy for us to determine. We may naturally fuppofe a perfon of Dr. F's turn for obfervation, reafoned from experience; and as his practice on thefe occafions might be attended with fuccefs, it feems by no means improbable that the cafes to which he refers were unattended with characteriftic marks of congeftion in the brain, otherwife I can hardly fuppofe he meant to difcard bleeding where real marks of plethora prefented.

Indeed, the too indifcriminate ufe of the lancet, adopted in almoft

* Vide Medical and Phyfical Journal, p. 562.

† Medical Transactions, Vol. i. p. 47.

‡ Vide Medical and Phyfical Journal, p. 562.

almoſt every caſe which had been ſuppoſed to be Apoplexy, particularly in thoſe where there appeared to be a ſuſpenſion or derangement in the vital organs, was, and ſtill is, too apt to be conſidered as ſuch; hence Dr. Heberden ſuſpected,* and after him Dr. F. was of opinion,† that this operation too frequently proved hurtful. I do not, however, infer from this, that the omiſſion of venæſection, or the exhibition of emetics in Apoplexy in general, are by any means ſafe or juſtifiable. In caſes that are ſtrongly marked, it is certainly proper to bleed *ad vires*; and as it is undoubtedly, at all events, hazardous to venture on the uſe of emetics, I ſhould certainly prefer that of evacuating by the inteſtines.

To me, however, it appears probable, though contrary to the notions of Dr. L. from whom I beg leave moſt reſpectfully to diſſent, that this very fit with which the lady was ſeized, was not occaſioned by *extravasation* of blood, *exudation*, or even effuſion, from the veſſels of the brain. Nothing ſeems more probable, than that it might have been brought on by a large indigeſted meal (which, as Dr. F.‡ obſerves) "diſtending the ſtomach, preſſing upon the aorta deſcendens, obſtructing the free expanſion of the lungs, and thus crowding the arterial ſyſtem of the head with more blood than ought to be there. Indeed, the good effects reſulting from the bleeding ſeems acknowledged, as the lady, after that, became *perfectly sensible.* It removed the turgeſcency or diſtention of the veſſels of the brain and lungs, which occaſioned compreſſion of thoſe parts. The treatment afterwards was proper and judicious; the opening medicine preſcribed by Dr. L. had operated prior to the leeches, which were not uſed till the day afterwards. The nauſea and ſickneſs at the ſtomach may, perhaps, be more rationally accounted for, from that part being irritated from the *indigestible* full meal than from the brain being primarily affected. Dr. L. certainly took the ſafeſt method by evacuating from the inteſtines; but admitting that the pulſe had become free, regular, and pretty uniform, after the bleeding, which is not noticed by either of the gentlemen, there is no great reaſon to ſuppoſe a gentle emetic might have proved hurtful, more eſpecially if the belly was not conſtipated. Be this as it may, it muſt however be allowed, that emetics on theſe occaſions are certainly hazardous.

Might not the pain, ſtricture, and ſenſe of oppreſſion which ſtill remained even after the operation of the purge, be owing to

* Medical Tranſactions, Vol. i. p. 473.

† Medical Obſervations and Inquiries, Vol. vi. p. 84.

‡ Idem Liber.

to a partial diftention, or local plethora, of the external veffels of the head, the immediate alleviation of which had been procured from the ufe of leeches? This, I think, appears probable, as the Doctor acknowledges, notwithftanding this, that the pulfe was tolerably free.*

Admitting that the mode of treatment adopted by Dr. L. in the cafe before us, as a phyfician, was perfectly right, and what may with fome little variation, according to circumftances, be applied generally with advantage in moft cafes of this fort; yet viewing him in the light of a Phyfiologift, one can hardly fuppofe him infallible, when he fo pofitively afferts that this cafe (which at any rate muft be confidered as doubtful) proceeded from actual *extravasation*, *exudation*, or *effusion* on the brain, and that fome one or more of thefe caufes muft exift as a *sine qua non*, to conftitute a *real* Apoplexy. Dr. L. muft pardon me when I affert, that very few, if any, converfant in diffections of this fort, will join him in that opinion.

Mr. C. when he contended that none of thefe caufes, he infifts, were *absolutely* neceffary to conftitute an Apoplexy, which might be produced by compreffion in the way as has been defcribed by Dr. F. was certainly not miftaken; and that this might be the cafe of the patient who is the fubject in difpute, is what I am almoft firmly perfuaded.

Dr. L. fays, (p. 559) "That all modern authors are *decided* upon this point, that compreffion is the only caufe of Apoplexy, and that it is produced by the effufion of blood, or ferum, and generally the former." In p. 560, he fays, "It is his firm belief that no cafe of *real Apoplexy* could poffibly occur without compreffion; and that when the fkull was not injured, the compreffion was *always* produced either by *extravasation*, *exudation*, or *effusion*, in *every instance*, when it amounted to abfolute abolition of the fenfes, and that the patient fell to the ground." Dr. Cullen, however, who is a *modern author*, and a favourite of Dr. L. in enumerating the appearances of the diffection of fubjects who have died of this difeafe, admits, among various other caufes, that it is occafioned "by blood accumulated in the blood veffels of the brain, and diftending them to fuch a degree as to comprefs the medullary portion of the fame."† The ingenious Dr. Kirkland confiders that fort of Apoplexy to which Dr. L. alludes, "to be a difeafe *sui generis*, arifing from an internal caufe, becaufe it has peculiar fymptoms; it does not always originate in the head, as has been imagined.

* Vide Medical and Phyfical Journal, p. 554.

† Vide Firft Lines, Vol. III. Art. 1103.

imagined, but alfo in the vifcera of the thorax or abdomen, or both together. It apparently happens to thofe poffeffing a morbid irritability, occafioned by fome kind of affection in the brain itfelf, or fome other part of the brainular fyftem."* The fame gentleman further obferves thus: "My friend, Mr. Chefshire, of Hinkley, a man to be depended upon, and whofe fkill in Anatomy enabled him to detect any morbid appearances, tells me he diffected many who died of Apoplexies in the Middlefex Hofpital, without being able to difcover any extravafation either of blood or ferum in the ventricles of the brain, or any part of the fkull." †

The celebrated Morgagni, in his elaborate Work entitled De fedibus et caufis Morborum, has noticed the fame. ‡

Moreover, the illuftrious and indefatigable Dr. Baillie, who is no lefs remarkable for his accuracy than the modefty and diffidence with which he advances his opinions on almoft every occafion, fays, "When blood is effufed into the fubftance of the brain, Apoplexy is produced, which is attended with the following fymptoms, viz. coma, often ftertorous breathing, a paralyfis commonly of one half of the body, and often convulfive motions; the pulfe is flow, full, and often very ftrong. When the patient is not cut off at once, but lives for fome time after the attack, the hemiplegia, which is almoft conftantly the effect of this difeafe, is upon the oppofite fide of the body from that of the brain, in which the effufion of blood has taken place." ||

From this quotation, it does not appear that Mr. C. was fo far miftaken as Dr. L. has taught us to believe, viz. "that in his judgment, if confiderable extravafation had taken place (in the cafe difputed) the moft formidable fymptoms muft have arifen;" or, in other words, he muft mean that more formidable appearances would have remained after the patient had recovered from the fit; and his faying, "there was no marks of paralyfis," which certainly was liable to have taken place, had this really been the cafe, appears, notwithftanding the Doctor's learned commentary, to be not altogether ill founded. Again, in Apoplexies to which pregnant women are fubject, my worthy preceptor, Dr. Denman, has obferved, that in the examination of thofe who have died, "I have never feen an inftance of effufion of blood in the brain, though the veffels were extremely turgid; but it is remarkable that, in all, the heart was found ufually

* Commentary on Apoplectic and Paralytic Effects, p. 17.

† Idem, p. 28.

‡ Vide Epift. 60. Art. 10. et feq.

|| Appendix to Morbid Anatomy, p. 161.

ufually flaccid, and without a fingle drop of blood in the auricles or ventricles.*

In the courfe of my practice, out of four cafes of puerperal apoplexies, three of them died. The firft, while in the act of parturition, fell back and fuddenly expired. The fecond remained in the fit feveral hours, but died half an hour after I was called in; fhe was the patient of another gentleman; venæfection and glyfter had been ufed, but in vain. The third, who previous to her attack laboured under anafarcous fwelling of the whole body, hard dry cough, dyfpnœa, &c. and who had been bled, and taken pectoral medicines without much benefit, was fuddenly feized with violent head-ach, ftertorous breathing, frothing at the mouth, fpafms of the mufcular fyftem, ftupor, and locked jaw, remained in this fit (before fhe quite recovered) fixteen hours, during which period fhe was bled freely from the arm, dafhings of cold water were alternately applied but with little fuccefs, and afterwards flannel cloaths wrung out of hot water, conftantly applied and repeated to the legs, thighs, and abdomen; acrid glyfters were alfo injected which procured ftools, and laftly a blifter inter fcapulas. She however recovered after the period of time mentioned, and the labour coming on gradually about twenty-four hours after, fhe was delivered on the 3d or 4th day of twins, one of which was dead. † The fourth patient was attacked in the fame way as the laft defcribed, with the very fame fymptoms, but more violent, her jaw being alfo clofed; the fame treatment was adopted in all refpects, but without fuccefs, fhe died in about four hours from the attack of the fit. Thefe three laft patients were in their firft pregnancy, the other had born children before.

None of the bodies of thefe women was I permitted to examine; but as it might have happened, had they been infpected that no extravafation, exudation, or effufion would have been, as appears to be the cafe with Dr. Denman's patients, yet I will not pretend to deny but their deaths might have occurred from fuch a caufe, as a cafe of this fort is related by Mr. Williams, of Rugley. ‡

I am however of opinion, that the third patient who recovered, and who is now living with her child, which is nearly four years old, might have had compreffion on the brain from the turgefcency of the veffels, but I never can believe it that there was extravafation.

Two

* Vide Introduction to the Practice of Midwifery, Vol. II. p. 408.

† This cafe, which is curious in fome refpects, is intended for publication in the Medical Tranfactions and Obfervations, by Dr. Simmons.

‡ Vide Medical Tranfactions and Obfervations, Vol. V. p. 96.

Two other cafes of Apoplexy have occurred to me in women advanced in life; their ages between fifty and fixty years; neither of them indicated the apoplectic *diathesis*, that is, they were not corpulent, fhort necked, or bloated, nor were they given to indolence or intemperance; both of them perfons in the lower ftation of life, both were dyfpeptic, and fubject to a fort of nervous irritability, and at times affected with head-ach, coftivenefs, &c. One of them had indulged herfelf in an afternoon with fome friends at a tea-drinking, where fhe freely indulged her appetite in eating heartily of cake kneaded with butter or hogs lard, and afterwards took a glafs of rum; foon after this, fhe was feized with violent head-ach, ficknefs at the ftomach, impeded refpiration, ftrong fpafms, her jaw clofed, and appeared quite fenfelefs: her pulfe felt remarkably flow, weak, and fluttering, and her countenance appeared pale, fo that I did not deem it proper to bleed her. Upon dafhing cold water upon her a few times, in two or three minutes, or lefs, fhe began to revive, vomited the contents of her ftomach, and had an involuntary loofe ftool, which greatly relieved her. A mixture Ex. magn. c. kali ppt. et conf. arom. was prefcribed; this, with fome tonic medicines, foon reftored her to her health. The other patient was feized nearly in the fame way with fimilar fymptoms, but more ftrongly marked, the fpafms being more violent, refpiration more convulfed, her jaw being alfo locked; the pulfe indicating fymptoms of plethora, being remarkably tenfe, I bled her *ad vires*, found her refpiration become more free and eafy; the convulfions appeared to go off, but fhe did not come out of the fit till I had ufed the dafhing of cold water, and then her fenfes began to return, and her jaw opened. Finding fhe had been much fatigued by over watching, want of reft, abftinence, and a little before the period of her attack had drank fome very acid hard ale, which had undoubtedly irritated her ftomach, acting as a fort of poifon, I prefcribed for her a fimilar mixture to that of the former patient, kali tartarizat. inftead of kali ppt. with good effect. It was, however, fome days before this patient recovered; fhe afterwards complained much of pain and heavinefs of her head, and had fymptoms of pyrexia. She took gentle laxatives, afterwards tonic medicines for a week, till fhe mended.

A remarkable ftout man, aged 63 years, pretty mufcular, with a large head, rather fhort neck, and full countenance, whofe occupation was fedentary, at certain times was affected with head-ach, ficknefs, indigeftion, and occafional coftivenefs. As he lived temperately, took the frequent exercife of walking in the open air, and kept his belly open with aloetic pills, &c. no medical advice was fought for till the beginning of June,

June, 1786, when I was confulted. At that time he laboured under great mental anxiety, with a dull heavy pain in his forehead and temples, accompanied with vertigo, depraved appetite, coftivenefs, pulfe full, and fomewhat oppreffed, with flight naufea, and thirft. The whole of thefe circumftances being well weighed as indicating an apoplectic *diathefis*, I bled him in the temporal artery, adminiftered an opening medicine, and he afterwards took fome dofes of James's powders, by which means his head became eafier, his thirft abated, and his pulfe pretty free, and regular: his mental anxiety, which was of the religious kind, ftill continuing, he became reftlefs, took but little food, and refufed all medical aid for two or three days. Being after this more compofed, he confented to take, as he was coftive, a purging medicine, Ex infus. fennæ. fal. Glaub. manna et tinct. jallapii, which was adminiftered in the morning. The whole of the forenoon he felt himfelf fick, with pain in his head and an inclination to vomit. As he attributed his indifpofition to the effect of the medicine more than to the increafe of his complaint, I was not fent for till he fell back fuddenly on the bed he was fitting, being then feized with a violent fit of what appeared to me to be a *real* Apoplexy. I found him totally fenfelefs, the convulfions were ftrong, the mufcular fyftem violently agitated, his breathing ftertorous, his jaw quite locked, his eyes ftaring and wild, with frothing at the mouth; a very copious involuntary difcharge by ftool had enfued, and his pulfe appeared funk. From all thefe appearances, and confidering that the fyftem had already been lowered from what had been done before, I was of opinion that a fatal extravafation, or effufion, (call it what you will) had taken place on the brain; and his jaw being clofed, fo that nothing could be given or got down, I deemed his cafe defperate, and left him. The time paft on, and I was fent for to fome other patients; but on enquiry long afterwards, found he was ftill alive and in the fit. It was then about 10 o'clock at night, when I again vifited him; he had been upwards of nine hours in this fituation, for the fit had come on about half paft 12 in the day. Finding his pulfe much more firm and fomewhat tenfe, I refolved to bleed him freely, whilft I reflected that it was *poffible*, *extravafation*, *exudation*, &c. might not have happened. At any rate no harm could enfue from the attempt. With fome difficulty, and a great deal of oppofition from thofe around me, as he lay on his back, ftill ftruggling, I bled him in the arm from a large orifice; the blood iffued out with freedom, and by the time I had filled a large bafon, I found him gradually recovering, his refpiration became more calm and eafy, the fpafms diminifhed, his jaw became relaxed, and his fenfes began to return. His pulfe now acting acting with freedom; as he had had a large evacuation by stool, I ventured to prescribe for him an anod. sudorific draught; this procured some sleep, with gentle diaphoresis, so that in the morning he appeared quite calm and sensible, the pain in his head, &c. being greatly lessened. By the time a fortnight had elapsed he seemed languid and much debilitated; his legs, thighs, face, hands, &c. appearing quite anasarcous, the secretion of urine was much diminished, his skin was dry, his belly costive, and pulse weakened. By the use of gentle saline cordial diuretics, together with mild purges, Ex pulv. crem. tart. c. pulv. arom. in forma elect. pretty strong infusions ex sem. sinapii et rad. raphn. rust. the anasarcous swellings went quite off, the secretion of urine even returned; and although he was supported by wine and jellies, was allowed some brandy and water, and such animal food as was easy of digestion, with the occasional use of tonic medicines, yet the integuments covering the whole of the external parts subject to the anasarcous affection never were restored to their tone, but continued loose and detached from their adhesion to the muscles.

As he had no cough, or difficulty in respiration, &c. it appeared that he was free from affections of the thoracic viscera; from a deficiency of bile, however, his stools, which were sometimes whitish, with high coloured urine, yellowness of the skin and tunica conjunctiva, and a sort of hardness and enlargement which appeared in the right hypochondrium, the liver appeared evidently diseased. His appetite, however, did not seem greatly impaired till the last, as he took his food pretty freely, and at times was in good spirits. In this way he continued till the middle of October, when he died. He did not keep his bed till about six weeks before, but got up and sat in his chair. No *paralysis*, *stupor*, or any disease indicating, or rather what might be expected from the effects of the fit to proceed from the brain, took place; extreme debility, and the return of the anasarca, combined in some degree with ascites, which appeared from the tension of the abdomen and swelling of the lower extremities, deficiency of the secretion of urine, aided by a partial mortification of the podex, nates, and part of the integuments covering the os sacrum, from the pressure of lying in bed, hastened his death, which took place after a period of near four months from his first illness. These three cases which we have related, among several others that I have *noted*, are adduced if not as absolutely positive, may yet be considered as presumptive proofs that apoplexy, or at least symptoms of apoplexy, occur independent of *extravasation*, &c. on the brain. Both of the women were, prior to the attack, in an apparent state

ſtate of tolerable health; they ſhewed no appearance of the apoplectic diatheſis, or tendency to ſuch a diſeaſe, and have ſince continued, and are now in a pretty good ſtate of health. The third, or laſt caſe deſcribed, wherein a tendency to this diſeaſe ſeemed to evince itſelf from external appearances, and where the fit laſted ſo many hours, ſhewed every appearance of this diſeaſe in the ſtrongeſt light; and although it can ſcarcely be doubted but Dr. L. will deem this caſe (if not the others) as being induced by *extravasation*, &c. yet, conſidering that the viſcera was even at that period in an incipient ſtate of diſeaſe prior to his attack, and no further appearances of Apoplexy, or its conſequences, having ever appeared after the acceſſion of his fit, I am of opinion that this Apoplexy proceeded more from a morbid ſtate of the viſcera inducing it, than from any primary affection that took place in the brain, and that it was not produced by *extravasation*, &c. No one however pretends to deny the poſſibility or even probability of extravaſated blood, or ſerum, independent of external injury, producing this diſeaſe; but whether this may be the cauſe or effect has been diſputed by ſome;* nor need we doubt that the former (I mean extra-vaſated blood) is ſometimes abſorbed by the powers of the ſyſ-tem, aided by evacuations, ſudorific opiates, &c. but this I apprehend will be found to be the caſe oftener in inſtances proceeding from external violence done to the head by accidents, and this even where no chirurgical operation took place.†.

Admitting, however, that abſorption of extravaſated blood occaſioning apoplectic ſymptoms, is effected in the firſt inſtance; it ſeems however doubtful that the effuſion, which is uſually of the ſerous kind, frequently remains behind. Dr. Baillie remarks that, "Cavities containing a ſerous fluid are ſometimes obſerved in the ſubſtance of the brain. They almoſt conſtantly occur in the medullary part of the hemiſpheres, and are generally lined with a tough ſubſtance or membrane. They would appear to be the remains of the cavities formed by extravaſated blood, in caſes of apoplexy where the patients have not been cut off immediately, but have lived afterwards for ſome months or years. The extravaſated blood would ſeem in ſuch caſes to be diſſolved, and taken up by abſorption; but the injury is not repaired, and a cavity remains afterwards filled with a ſerous fluid."* After what has already been ſaid relative to the dif-

* Vide Kirkland on Apoplexy. Morgagni Epiſt. 4to. &c.

† Bromfield's Obſervations in Surgery. Pott's Works, Bell's Syſtem of Surgery, &c.

* Appendix to the Morbid Anatomy, p. 151.

ferent opinions of Mr. C. and Dr. L. in this cafe, as profeffional men, in which it appears that my fentiments differ widely from the latter gentleman's, it might perhaps have been expected by fome of my readers, that I fhould have thrown out fome new light on this uncertain, difficult, and, at any rate, obfcure difeafe; but to this tafk I feel myfelf totally unequal. Neither fhall I prefume, after what has already been written on this very interefting fubject by thofe illuftrious characters who have preceded me, and whofe opportunities of invefti-gating this important difeafe have been greatly fuperior to mine, even to attempt to enter into a difquifition of the feveral varieties and diftinctions into which it has been divided; this, befides leading me into a labyrinth of mere fyftematic conjectures, would not in my opinion tend to produce any thing new or interefting on a fubject that has not hitherto been done by men of fuperior abilities; nor fhall I attempt to purfue Dr. L. any further in his definitions of fanguineous and ferous apoplexy, and his animadverfions on the different doctrines and practice of thofe who have preceded him. Thefe fhall be left or fubmitted to the profeffion at large, who are beft enabled to judge how far Dr. L. from the fpecimen he has now exhibited before us of his phyfiological talents, may be qualified to perform what he has promifed to lay before the public at a future time, and which, I truft, after what has happened, he will not difappoint; his words are thefe. "And it is now my determination, from feeing the danger of defective difcrimination in *these cases*, *the moment I have a little leisure*, to write upon this fubject in fuch plain and intelligible terms, as to enable the *young and most inexperienced practitioner*, when called upon in fuch emergencies, to act with promptnefs and decifion, and with confidence in himfelf, till more experienced affiftance can be procured, as a few minutes delay, in fuch inftances, might prove fatal."* This, it muft be owned, is a fplendid promife; and if it were not fubject to the exception of, "the moment I have a little leifure," I entertain no doubt that *the most experienced*, not to mention *inexperienced*, practitioners will acknowledge themfelves highly indebted to him for this happy and unlooked for difcovery. From the firft moment of my perufing thefe different ftatements, I felt my mind impreffed with the difficulty of the fubject in difpute, with which it is connected, and which it muft be allowed is certainly of the utmoft importance, not only by reafon of its doubtful nature, which has given rife to different opinions of its caufe, but that of the various

* Vide Medical and Phyfical Journal, No. xxxiv. p. 561.

various modes of treatment which have been deemed neceſſary to adopt. This, by inducing me to turn over in my mind what had actually fallen under my own immediate obſervation, and comparing it with what has already been advanced on this ſubject by the moſt approved modern writers of the preſent day, (to whom Dr. L. ſeems principally to appeal) together with the hopes and expectations of its ſtill being further elucidated by more able hands, is a principal cauſe of my preſuming thus to obtrude my ſentiments upon the public. To neither of the parties concerned in this diſpute am I known perſonally, nor to their private animoſities do I pay the leaſt regard whatever; and how far I may have acted with candour and impartiality in the diſpute between them, is ſubmited to the conſideration of impartial profeſſional readers. I am, &c.

*Sunderland, Dec.* 21, 1801. G. WILKINSON.

---

### *To the Editors of the Medical and Phyſical Journal.*

GENTLEMEN,

