# Errata

**Published:** 1802-03

**Authors:** 


### ERRATA.

Vol. vii. p. 142, Note *, *for* effects *read* affections.

143, at the bottom, *for* Med. Tranſactions, *read* Med. Facts.

——, line 2, from the bottom, after believe, *dele* it.

——, line 6, from the bottom, *for* Rugley, *read* Rugby.

——, line 8, *for* glyſter, *read* glyſters.

146, line 23, after ſtools, *dele* which.

148, line 11, *for* even to attempt, *read* even attempt.

